# The Safety and Pharmacokinetics of Carprofen, Flunixin and Phenylbutazone in the Cape Vulture (*Gyps coprotheres*) following Oral Exposure

**DOI:** 10.1371/journal.pone.0141419

**Published:** 2015-10-29

**Authors:** Tamsyn Fourie, Duncan Cromarty, Neil Duncan, Kerri Wolter, Vinny Naidoo

**Affiliations:** 1 Department of Paraclinical Sciences, Faculty of Veterinary Science, University of Pretoria, Pretoria, South Africa; 2 Department of Pharmacology, Faculty of Health Sciences, University of Pretoria, Pretoria, South Africa; 3 Vulture Programme (VulPro), Plot 121, Rietfontein, 0048, South Africa; Justus-Liebeig University Giessen, GERMANY

## Abstract

The following study evaluates the overt toxic potential of carprofen (CRP), flunixin (FXN) and phenylbutazone (PBZ) in Old world vultures in relation to historic toxicity data for diclofenac and ketoprofen, with the Cape vulture (*Gyps coprotheres*) being the indicator species. The toxic potential of a single oral dose of CRP (11.5 mg/kg), FXN (1 mg/kg),PBZ (1.7 mg/kg) or water was evaluated by means of a four-way parallel study (n = 2), as means of ascertaining if these drugs were as toxic as diclofenac in the vulture. No unscheduled deaths or pathological lesions were noted following exposure. Clinical signs of lethargy and depression were, however, noted in one CRP, two FXN and one PBZ treated birds. Mild reversible inhibition of UA excretion was evident in all three groups, although UA remained within the population reference interval in contrast to the effects previously described for diclofenac and ketoprofen. All treatment groups had a drug concentration responsive increase in alanine transferase activity. CRP, FXN and PBZ were characterised by a maximum plasma concentration (Cmax) of 1051.8 ± 620.7 ng/ml, 335.9 ± 36.3 ng/ml and 11150 ± 2474.9 ng/ml at 4 ± 4.3, 0.45 ± 0.02 and 5.3 ± 5.2 hours (Tmax) respectively and a half-life of elimination of 13.3 ±5, 1.8±1 and 18.7 ±11.4 hours respectively. While we could not demonstrate a lethal effect of the tested substances, the presence of toxic clinical signs, clinical pathological changes and/or long half-lives of elimination suggests that all three drugs have a potential for toxicity in a larger population or on repeat administration. In conclusion while the studied substances were not as overtly toxic as diclofenac, they are of safety concern.

## Introduction

Vultures are an integral part of the ecosystem as they are important in clearing dead carcasses in a short period of time (in less than an hour in some cases) [1]. The loss of these valuable keystone species could be catastrophic to any ecosystem. Unfortunately, at present this is becoming a reality for many vulture species across the world. The same is the case in South Africa, as seven of the nine species found in the region are listed as endangered, vulnerable or critically endangered [2], with declines having been attributed to numerous causes such as poisonings (intentional persecution and accidental) [3]; loss of habitat and loss of available safe food [3]; electrocutions and collisions on electricity pylons [4]; harvesting for traditional medicines [5], drowning in farm reservoirs [6], disturbances at breeding/roosting sites and direct persecution due to a lack of education and knowledge.

Various mitigating measures have been implemented to minimise these impacts on the population numbers such as supplementary feeding, community awareness programmes, insulation of the conductors, moving tower cross arms, providing alternate perches and the fitting of reservoirs with floaters/nets [6–8]. Further measures have included the treatment of injured birds for later release. However, it is not possible to mitigate against every scenario, as seen in recent years with the tragic demise of large populations of Gyps vultures in Asia due to the exposure to diclofenac, that inadvertently entered into their food chain [9,10]. With the wide scale seemingly safe use of diclofenac in both human and veterinary medicine, (having fairly typical side effects that are rarely fatal), it was completely unexpected that diclofenac could be so lethal to vultures. More recently studies have demonstrated that the entire class of the non-steroidal anti-inflammatory drugs (NSAIDs) appears to be unpredictable in their toxicity in the Cape vulture (*Gyps coprotheres*) with ketoprofen being similar to diclofenac [11,12] whilst meloxicam was safe [13]. Most recently, a dead griffon vulture (*Gyps fulvus*) found with nephrotoxicity and flunixin (FXN) tends to suggest that FXN is also toxic to Old world vultures [14].

With numerous other NSAIDS being available globally for veterinary use, the safety of these drugs has been questioned. With *in-vivo* toxicity testing being the only predictive method thus far available, the following study will add to the overall knowledge of the NSAID’s FXN, phenylbutazone (PBZ) and carprofen (CRP) in the Cape vulture, using the same study design and sample size (n = 2) previously validated to demonstrate equivalent toxic effects to diclofenac.

## Materials and Methods

### Treatment of animals and ethics statement

Non-releasable captive Cape vultures (n = 8) (Table 1) were used in the study and exposed to the test drug or water in pairs as for the previous diclofenac toxicity studies [15,16]. Ethical considerations for the study were approved by the Animal Use and Care Committee of the University of Pretoria (Protocol Number: V006-10) and research on an endangered species was approved by the relevant South African Department of Nature Conservation. The bird pairs were housed in aviaries of 5 x 3 x 3 m in size, with soil floors, perches and diamond mesh sides under natural environmental conditions. The doses for FXN (Finadyne, Scherring-Plough) and PBZ (Fenylbutazone, Virbac), were determined as twice the maximum tissue concentration from cattle, horses or pigs. For PBZ this was the horse kidney at 3.4 mg/kg of kidney and FXN in cattle liver at 1.95 mg/kg of liver [17,18]. With the intake of food estimated at 0.52 kg at a feeding for an 8 kg bird [19], the dose of FXN and PBZ was 1 and 1.7 mg/kg respectively. For CRP (Rimadyl, Pfizer) which also has potential veterinary use, the birds were exposed to the recommended raptor dose of 10 mg/kg [20].

**Table 1:** Details of the birds inducted into the study.

Bird	Tag Number	Group	Age (years)	Weight (kg)	Sex
1	G24190	Carprofen	<1	7.2	Female
2	G30492	Carprofen	1–3	9	Female
3	G30474	Flunixin	1–3	7	Female
4	G30418	Flunixin	1–3	9	Female
5	G30969	Phenylbutazone	1–3	7.2	Female
6	G30086	Phenylbutazone	2–4	8	Female
7	G27048	Control	3–5	8	Female
8	G30430	Control	1–3	9.2	Female

### Observations

Animals were frequently observed over 48 hours for clinical signs of toxicity. Blood samples were also collected from the tarsal vein (or when necessary the wing vein or jugular vein was used) immediately before drug administration and at 5 and 30 min; 1, 1.5, 2, 3, 5, 7, 9, 12, 24, 32 and 48 hours after treatment with a 21G needle and 5 ml syringe. Half the sample was placed into either an EDTA or anti-coagulant free evacuated tube (Vacutainer, Becton Dickinson, South Africa) and allowed to separate. The plasma or serum samples were banked at -20°C for up to two years until analysed. Serum samples were analysed by the Department of Companion Animal Studies, Clinical Pathology Laboratory, University of Pretoria, two years after collection using the Cobas Integra 400 (Roche Diagnostics) for activities of alanine transferase (ALT), concentrations of albumin (ALB), calcium (Ca), potassium (K), sodium (Na) and uric acid (UA). Changes in the measured clinical pathology parameters were considered significant if the changes were; different to the control group; outside of the published population reference interval [16]; or were different to the bird’s baseline values.

Plasma samples were analysed by the Department of Pharmacology, University of Pretoria for their PBZ, FXN or CRP concentration using validated LC-MSMS methods (For details of the method see S1 to S3 Methods and S1 to S3 Figs). Following the 48 hour monitoring period, animals underwent euthanasia with intravenous sodium pentobarbitone (Euthapent, Bayer) for post mortem evaluation. Samples collected from parenchymatous organs in 10% buffered formalin were evaluated for histopathological changes following examination with H&E staining.

### Pharmacokinetic analysis

The pharmacokinetic analysis was undertaken in Kinetica 5.2 (ThermoElectron Corporation) using non-compartmental modelling. The terminal phase was utilised to determine the elimination half-life (T½) and elimination rate constant (λ). The area under the plasma concentration versus time curve (AUC) and the area under the moment curve (AUMC) was obtained using the linear trapezoidal rule, up until the last measurable concentration (C_last_), with extrapolation to infinity (AUC_∞_) using the elimination rate constant (C_last_/λ). Total body clearance (Cl = dose/AUClast), the apparent volume of distribution (Vz/F = Dose/AUC* λ) and the mean residence time (MRT = AUMC/AUC) were calculated using standard formulae. Plasma drug concentrations were also plotted against their corresponding ALT activity or UA concentration, to ascertain the relation of changes in these variables with changes in plasma concentration of drug. Graphs were also evaluated for irreversible changes which were not linked to changes in plasma concentrations. Previously collected data for diclofenac, ketoprofen and meloxicam, were also analysed for comparative purposes when available [11–13,16].

## Results

After dilution of the study drug the actual doses administered were 11.5, 1 and 1.7 mg/kg for CRP, FXN and PBZ respectively. No mortalities were recorded following treatment. Clinical signs of lethargy and depression were noted in the 1 CRP treated bird, 2 FXN treated birds, and 1 PBZ treated bird while none were reported for the control birds. The depression in all cases had resolved by 48 hours post exposure. All necropsies were unremarkable, with no signs of urate crystals deposition or tophi formation. Histopathological evaluation confirmed no signs of pathology, except for some scattered renal tubules showing some signs of pyknosis and karryorhexis with no granular or cellular casts.

**Fig 1:**
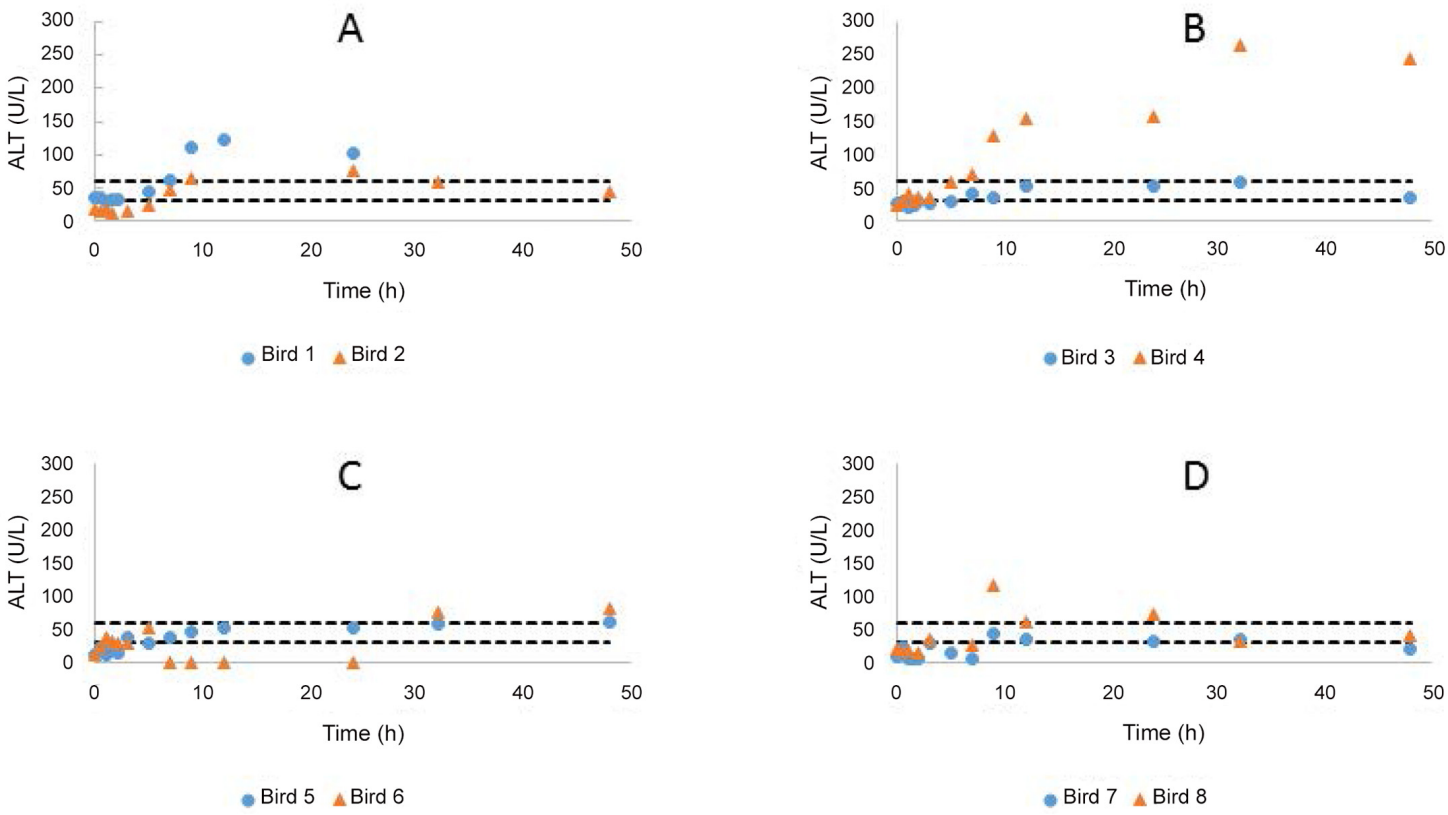
Changes in ALT activity over time for the individual birds treated with carprofen (A), Flunixin (B), Phenylbutazone (C) in comparison to the controls (D). The dotted lines represent the population upper and lower tolerance.

**Fig 2:**
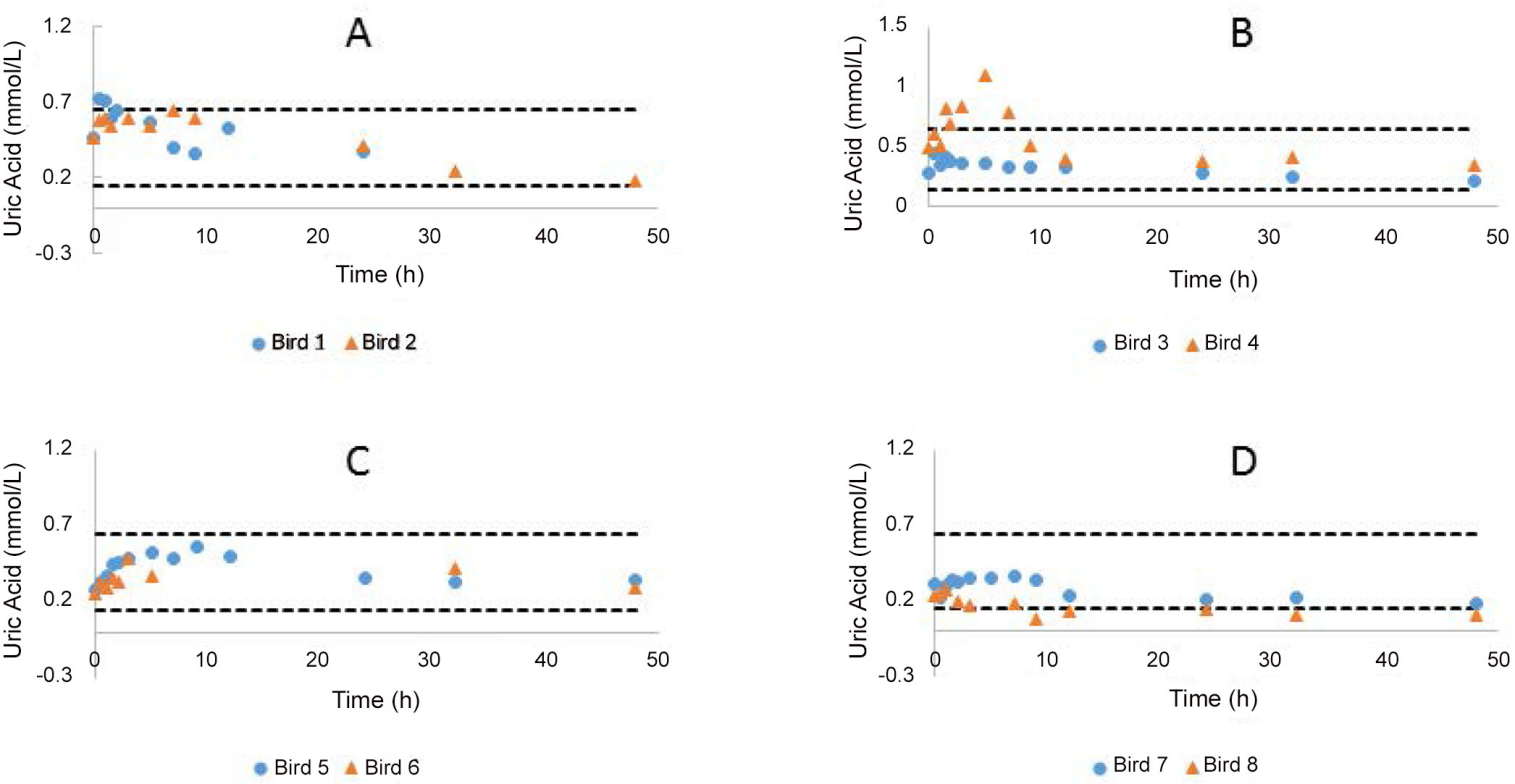
Changes in uric acid concentration for the individual birds treated with carprofen (A), flunixin (B), phenylbutazone (C) in comparison to the controls (D). The dotted lines represent the population upper and lower tolerance.

Numerous changes were seen in the evaluated clinical pathology parameters over time (S1 to S6 Tables). The changes for ALB, Na, K and Ca were not considered significant as changes were either similar to the controls or within the population reference intervals. More pronounced changes were seen with ALT activity (Fig 1), an important marker of diclofenac induced toxicity in vultures. The CRP treated birds showed a gradual increase in ALT activity which peaked (average 4.6 fold higher than pre-treatment) at 12 and 24 h before declining. One FXN treated bird showed increasing ALT activity up to 32 hours. The FXN treated birds had an average ALT activity above population reference intervals after 9 hours, which peaked at 32 hours (6.2 fold higher than pre-treatment in FXN treated birds). Both the PBZ treated birds demonstrated a steady increase in ALT serum activity, which failed to peak at the last sampling point (4.8 and 6.4 fold greater than pre-treatment). Uric acid concentration remained within the population reference interval except for one bird in each of the CRP and FXN groups (Fig 2). For the CRP bird the increase in UA was marginal over the reference interval while the change for the FXN bird was more substantial (1.7 fold greater than the population reference interval). Both the birds that demonstrated a change in UA concentrations, were the ones that had the greatest change in plasma ALT activity.

**Fig 3:**
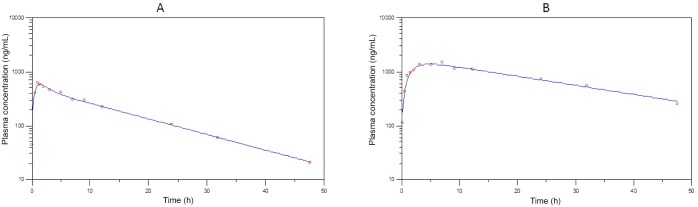
Plasma concentration versus time after dosing, plot (dots) with best fit curve (lines) for bird 1 (A) and 2 (B) following oral administration of carprofen (11.5 mg/kg). Samples were collected sequentially, when possible, at 5 and 30 min; 1, 1.5, 2, 3, 5, 7, 9, 12, 24, 32 and 48 hours after treatment with carprofen.

**Fig 4:**
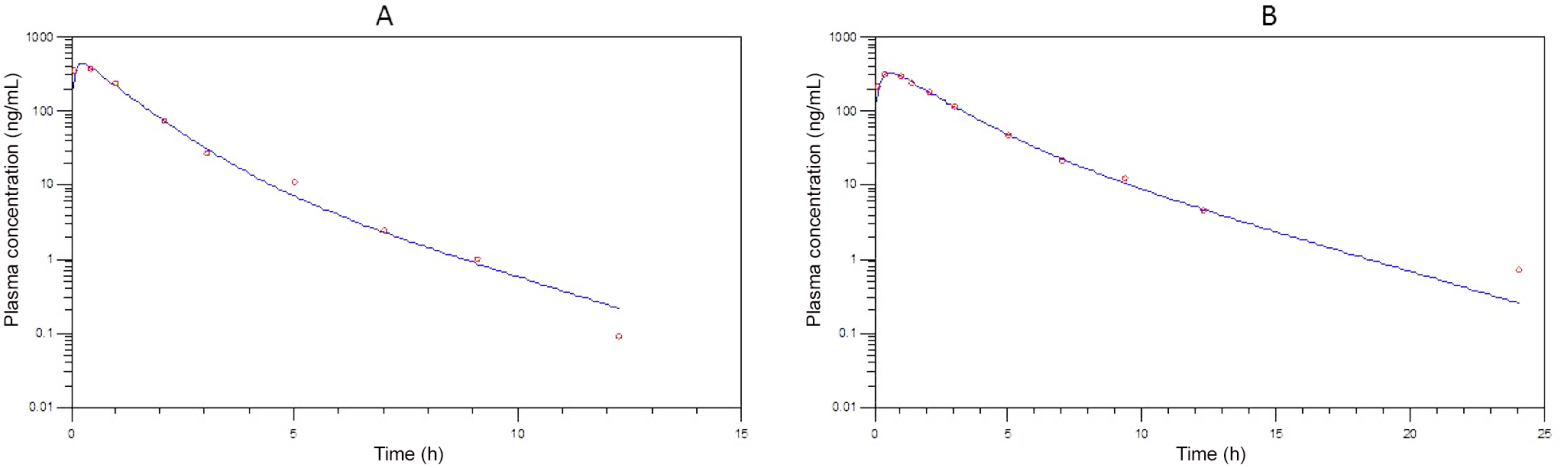
Plasma concentration versus time after dosing plot (dots) with best fit curve (lines) bird 1 (A) and 2 (B) following oral administration of flunixin at 1 mg/kg. Samples were collected sequentially, when possible, at 5 and 30 min; 1, 1.5, 2, 3, 5, 7, 9, 12, 24, 32 and 48 hours after treatment with flunixin.

**Fig 5:**
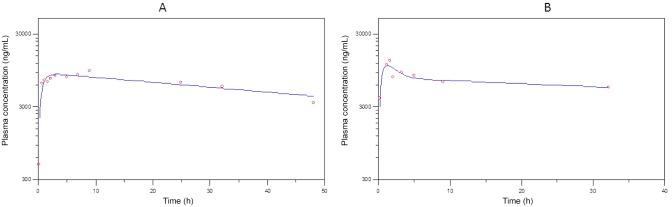
Plasma concentration versus time after dosing plot (dots) with best fit curve (lines) bird 1 (A) and 2 (B) following oral administration of phenylbutazone at 1.7 mg/kg. Samples were collected sequentially, when possible, at 5 and 30 min; 1, 1.5, 2, 3, 5, 7, 9, 12, 24, 32 and 48 hours after treatment with phenylbutazone.

The pharmacokinetic curve fitted plots are presented in Figs 3 to 5 and the pivotal non-compartmental parameters are presented in Table 2. Oral absorption of CRP, FXN and PBZ was characterised by a maximum plasma concentration of 1051.8 ± 620.7 ng/ml, 335.9 ± 36.3 ng/ml and 11150 ± 2474.9 ng/ml obtained in 4 ± 4.3, 0.45 ± 0.02 & 5.3 ± 5.2 hours respectively with a corresponding elimination half-life of 13.3 ±5, 1.8±1 & 18.7 ±11.4 hours respectively. Volume of distribution was 13.62 ± 9.91 L/kg; 3.29± 0.75 L/kg & 0.13 ± 0.03 L/kg for CRP, FXN and PBZ respectively. Area under the curve until the last time point was 21.72± 20.1; 0.78± 0.28 & 263.35 ±68.69 μg/mL*h for CRP, FXN and PBZ respectively.

**Table 2:** Non-compartmental parameters for carprofen, flunixin and phenylbutazone.

PK Parameter	Unit	Carprofen			Flunixin			Phenybutazone		
		Bird 1	Bird 2	Ave.	Bird 3	Bird 4	Ave.	Bird 5	Bird 6	Ave.
**Cmax**	**ng/mL**	612.87	1490.68	1051.78	361.59	310.2	335.89	9400	12900	11150
**Tmax**	**h**	1	7.08	4.04	0.47	0.43	0.45	9	1.62	5.31
**AUClast**	μg/mL*h	7.51	35.93	21.72	0.58	0.98	0.78	311.92	214.78	263.35
**AUCextra**	μg/mL*h	0.27	6.35	3.31	0	0	0	136.28	21.72	79
**AUC**_**∞**_	μg/mL*h	7.78	42.28	25.03	0.58	0.98	0.78	448.21	236.49	342.35
**%AUCextra**	**%**	3.427	3.43	15.01	0.03	0.02	0.02	30.407	30.41	9.18
**λ**	**1/h**	0.07	0.04	0.06	0.63	0.27	0.45	0.03	0.07	0.05
**t½**	**h**	9.67	16.86	13.26	1.1	2.58	1.84	26.79	10.64	18.72
**MRT**	**h**	14	25.81	19.9	1.24	3.1	2.17	40.47	17.51	28.99
**Clearance**	L/h*kg	1.48	0.27	0.88	1.74	1.02	1.38	0.004	0.007	0.005
**Vz/F**	**L/kg**	20.63	6.61	13.62	2.75	3.82	3.29	0.15	0.11	0.13

Cmax—Maximum plasma concentration;

Tmax—Time to maximum plasma concentration;

AUClast—Area under the plasma concentration versus time curve to the last quantifiable time point;

AUCextra—The are under the plasma concentration versus time curve extrapolated from the last time point to infinity;

AUC_**∞**_- The sum of AUClast and AUCextra;

%AUCextra—The percentage of AUC_**∞**_ **value represented by** AUCextra;

**λ**- The elimination constant;

t½- Half-life of elimination;

MRT-Mean residence time,

Vz/F- Apparent volume of distribution.

## Discussion

This study attempted to ascertain the overt acute toxicity of CRP, FXN and PBZ using the Cape vulture as an indicator species for other Old world vultures due to their equivalent susceptibility to diclofenac as the white-rumped vulture (G. bengalensis), African white-backed vulture (G. africanus) and griffon vulture (G. fulvus). For this study, birds were exposed to CRP, FXN and PBZ in groups of two as per the study design specified by Cuthbert et al (2006) [21]. In their calculation of an adequate sample size for endangered population of vultures, Cuthbert indicated that this small sample size was of statistical significance when ascertaining if a substance was as toxic as diclofenac, while larger sample sizes (n > 40) would be needed for sufficient statistical power to ascertain if a substance was of lower toxicity. From a exposure point, this study is therefore statistically valid in providing evidence of the ability of the study drugs to induce death in vulture in a similar manner as diclofenac, while at the same time being wholly insufficient to indicate if the drug would be absent of minor toxic signs, side effects or induce lower incidence of mortality. The major advantage of this sample size is its ability to ascertain the presence or absence of severe toxic potential without requiring the death of a large number of an endangered species, as would be required if a standard preclinical study design was to be used.

The exposure doses used for this study are also considered to be realistic as they were based on either a worst case scenario of the birds being exposed to high tissue concentration of the drug in recently dead cattle, horses or pigs which would represent their predominant food source (FXN and PBZ), or potential higher veterinary exposure (CRP). The reason for the latter is that CRP together with ketoprofen and meloxicam are recommended as analgesics support in raptors [20]. With ketoprofen being a known toxin from this list, we felt it safer to consider the toxicity of CRP from a point of veterinary use as well as from exposure through the diet [22]. Using the same methodology as for FXN and PBZ, the CRP dose chosen for this study represents a threefold higher exposure than would be expected in the meat (3.32 mg/kg in horse meat). With no deaths evident following oral exposure, it is concluded that the three NSAIDs evaluated are not as toxic as diclofenac and ketoprofen in vultures. This conclusion can be drawn as the same study design was followed as that of Swan et al (2006) and Naidoo et al (2009) who previously evaluated diclofenac in Old world vulture species [15,16].

The absence of overt toxicity for all three study drugs was an unexpected finding as the questionnaire based survey of Cuthbert et al (2007), indicated that these drugs were associated with toxicity in numerous Old world vultures [23]. The study result for CRP was, however, similar to that for the domestic fowl (*Gallus gallus domesticus*) at 0.3 to 40 mg/kg and for the Hispaniola parrots (*Amazona ventralis*) at 3 mg/kg for which no toxicity was evident [24–26]. For FXN the drug appeared to be less toxic in the Cape vulture than in the Siberian cranes (*Grus leucogeranus*), whooping cranes (*G*. *americana*), red-crowned cranes (*G*. *japonensis*) which showed signs of renal ischemia, necrosis and gout at 5 mg/kg [27]; the bobwhite quails (*Colinus virginianus*) which showed signs of renal toxicity at doses as low as 0.1 mg/kg [28] and the griffon vulture for which toxicity was linked to consumption of contaminated meat (no dose given) [14]. The absence of toxicity from PBZ was not completely unexpected as very high doses, 50 mg/kg and 100 mg/kg administered intramuscularly twice a day to broilers, was required for toxic signs to be seen [29].

Nonetheless despite the absence of overt acute toxicity, we emphasise that these drugs cannot be concluded as being comparatively safe to meloxicam [30]. The major reason is the need for a much larger sample size closer to 40 to demonstrate a molecule’s less obvious toxic potential, as evident with ketoprofen and meloxicam which required much larger sample sizes to elucidate or refute their toxic potential respectively [12,13,31]. Other reasons for this conclusion, are based on the presence of other indicators of toxicity such as the clinical signs induced, changes in clinical pathology and pharmacokinetic variables calculated. From the clinical signs, various birds showed a degree of depression and drooping head, which was previously seen in diclofenac and ketoprofen exposed birds [10–12, 15, 16].

Changes in ALT activities and serum UA concentrations were also present for all the test drugs. In mammalian species, ALT is an enzyme found in the cytosol of hepatocellular cells. When acute hepatocellular injury results, these enzymes leak into the vasculature, increasing serum enzyme activity [32]. ALT, bound to the plasma membrane, can also be increased in states of enzyme induction e.g. drug / hormonal effects [32]. Although ALT activity in many mammalian species is indicative of hepatocellular damage, in psittacine species serum ALT activity may increase due to damage to the liver, heart, skeletal muscle, lung or intestine [33]. In contrast to many mammalian species, the highest tissue distribution of ALT was found in the kidney of domestic fowls [34]. As a result it would appear for the majority of the drugs, the changes induced at the cellular level were minor and reversible and more importantly were not serious enough to be histologically evident. However with a single dose having the ability to induce some change in the parameter, it is likely that repeat doses or higher doses could induce more severe lesions. The magnitude of increase of ALT activity in the one FXN bird is however harder to explain. We’re currently uncertain as to why the ALT activity was increased without corresponding pathology being present. In a previous study in which two Cape vultures were exposed to diclofenac, a tenfold increase in ALT activity and severe liver and kidney pathology were present 48 hours after dosing [15]. With the absence of evident hepatic or renal pathology on histological evaluation, one possible explanation would be that the bird in question may still have succumbed to toxicity if the study was not terminated at 48 hours for necropsy evaluation.

Uric acid concentrations were specifically monitored as it was an important indicative parameter for toxic changes in both ketoprofen and diclofenac toxicity. All the treated birds in this study showed an increase in UA concentration, which decreased to pre-treatment concentrations by 48 hours, without concurrent histological lesions of renal damage or changes in serum potassium concentrations. This increase in UA concentration (i.e. mild inhibitory effect on UA excretion) for PBZ was an unexpected finding, as it has been reported as having uricosuric activity in humans [35], although increased UA concentration has been noted in chickens [36]. The change in UA concentration, in general, was also within the population reference interval for the monitoring period. As a result it is concluded that the raised UA is more likely from reversible inhibition of UA excretion and not toxicity. The latter has been well described in human literature whereby the NSAIDs are known to interact with tubular UA transporters in a reversible manner [37].

One potential shortcoming of the study was the use of banked samples. While it may be argued that sample degradation could have resulted, it must be taken into consideration that other studies, albeit for human samples, have shown that serum stored at -25°C for 2 years can still produce reliable results [38,39] for Na, Ca, UA, ALB and K however not for ALT. It is however believed that for this study, the ALT activities are relevant, as the presence of the control group, pre-treatment controls from each bird and the evaluation of sequential changes over time for the monitoring period, provides for proper interpretation of the data. In addition, the increases in ALT activities seen were substantial when it did result.

A few important findings were present from the pharmacokinetic profiles that are also suggestive of toxicity either due to population variation or a cumulative drug effect. The pharmacokinetics of CRP differed with bird 2 having a seven fold increase in T_max_, double the C_max_, fivefold greater AUC_last_, and concurrent longer MRT and T_1/2_. This bird also showed signs of depression. A similar trend was present for the PBZ treated birds with the T_max_, AUC_last_ and T_1/2_ being larger for bird 5. With the grouping of birds known to have received the same dose, reasons for this difference could be slower gastro-intestinal transit time which while slowing down rate of absorption would result in a net greater extent of absorption. Another possible reason may be individual variability in metabolic capacity which would be similar to that seen for the vultures treated with ketoprofen [11].

The elimination half-life of CRP and PBZ were both long and more than 12 h, while FXN was characterised by a relatively short half-life. The half-life of CRP was not dissimilar to that described in the horse at 18.1 h [40], but was larger than the range of 3.2 to 11.77 h reported in the dog [41]. While we consider the half-life of PBZ, to be long, it should be noted that this was substantially shorter than the half-life of 70h for people [42] and 62.6 h for Holstein cattle [43] when administered orally. Flunixin’s half-life was similar to that described in other birds at, 0.62 h, 0.43 h, 0.54 h and 0.17 h for, pigeon, mallard duck, turkey & ostrich respectively [44] and at 0.72 h and 0.91 h for budgerigars & conures [45]. Based on the half-lives, FXN (1.84 h) is the least likely to accumulate on repeat administration while both CRP and PBZ (13.2 and 18.7 h) could result in toxicity with repeat administration. In order for toxicity to occur following repeat administration of CRP and PBZ to occur, the birds would need to feed within 66 hours (CRP) or 93.5 hours (PBZ) of the initial drug intake from another contaminated carcass. At feeding sites in South Africa and during the breeding season, vultures have been noted to feed daily (K Wolter, Per Comm). Repeat exposure is therefore a possibility if the prevalence of residues in carcasses is high.

## Conclusion

From the specific study design used, it was concluded that CRP, PBZ and FXN are not as toxic to vultures as diclofenac. We are unable to conclude on the general safety of these tested drugs, as they all show some indication towards toxicity. As such a larger exposure study, similar to that of meloxicam, needs to be implemented.

## Supporting Information

S1 Fig Calibration curve for carprofen, blue line indicating the average of 3 runs and error bars (±1 SD). (TIF)

S2 Fig The calibration curve for flunixin, the blue line indicating an average of 3runs and error bars (±1 SD). (TIF)

S3 Fig The calibration curve for PBZ, the blue line indicating an average of 3 runs. (TIF)

S1 Method Quantification of drug in the plasma. (DOCX)

S2 Method Carprofen & Flunixin. (DOCX)

S3 Method Phenylbutazone analysis. (DOCX)

S1 Table Mean and standard deviation (SD) of the serum ALB concentrations (g/L) per treatment group per time of sampling. (DOCX)

S2 Table Mean and standard deviation (SD) of the serum activities of ALT (U/L) per treatment group per time of sampling. (DOCX)

S3 Table Mean and standard deviation (SD) of the serum Ca2+ concentrations (mmol/l) per treatment group per time of sampling. (DOCX)

S4 Table Mean and standard deviation (SD) of the serum Na concentrations (mmol/l) per treatment group per time of sampling. (DOCX)

S5 Table Mean and standard deviation (SD) of the serum K^+^ concentrations (mmol/l) per treatment group per time of sampling. (DOCX)

S6 Table Mean and standard deviation (SD) of the serum UA concentrations (mmol/l) per treatment group per time of sampling. (DOCX)
