# Supplementary figures and images for: The Safety and Pharmacokinetics of Carprofen, Flunixin and Phenylbutazone in the Cape Vulture (Gyps coprotheres) following Oral Exposure
Source: PLoS One. 2015 Oct 29;10(10):e0141419. doi: 10.1371/journal.pone.0141419 (PMC4626400; doi:10.1371/journal.pone.0141419)

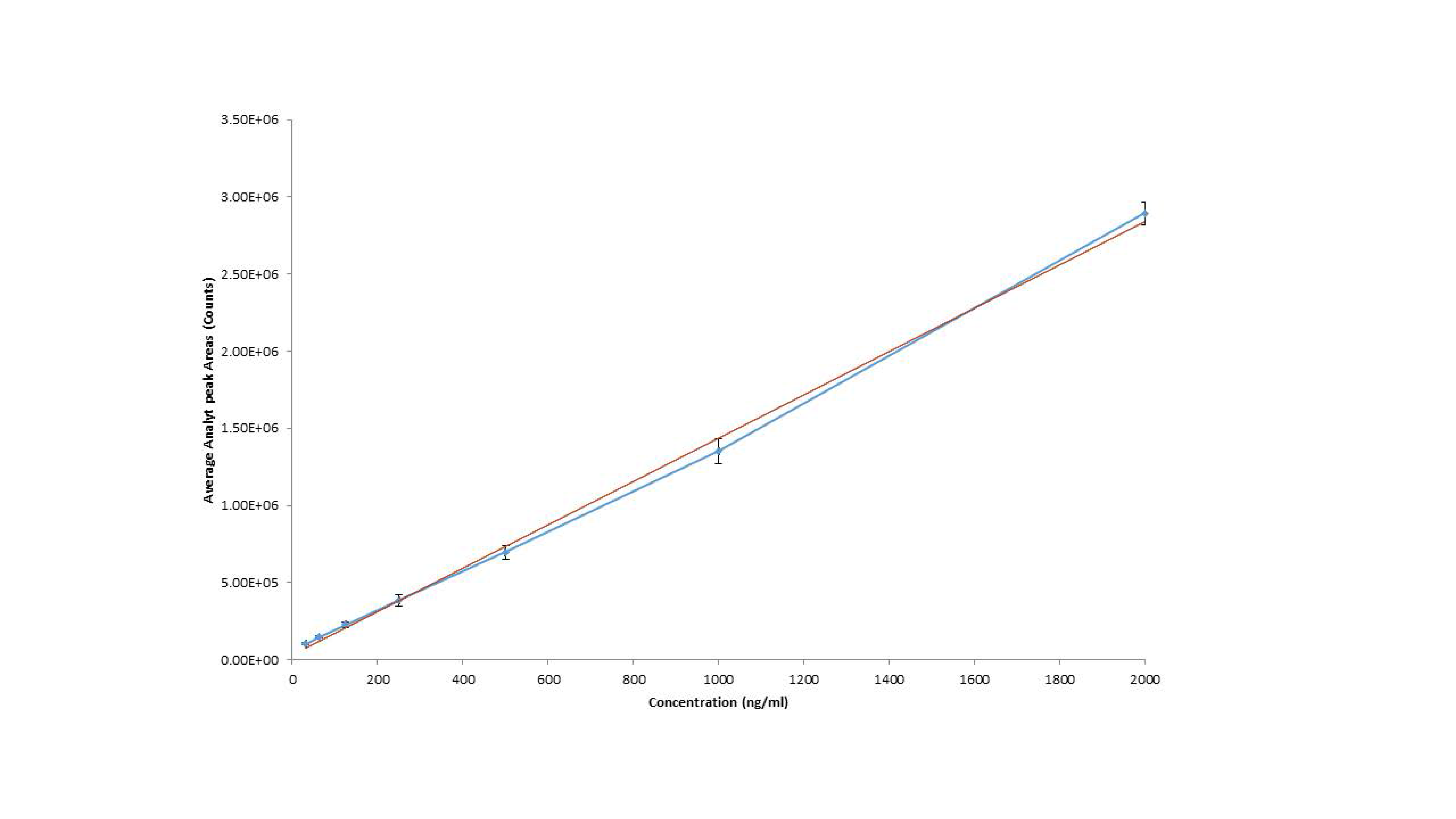

Supplement: S1 Fig — (TIF) [file pone.0141419.s001.tif]

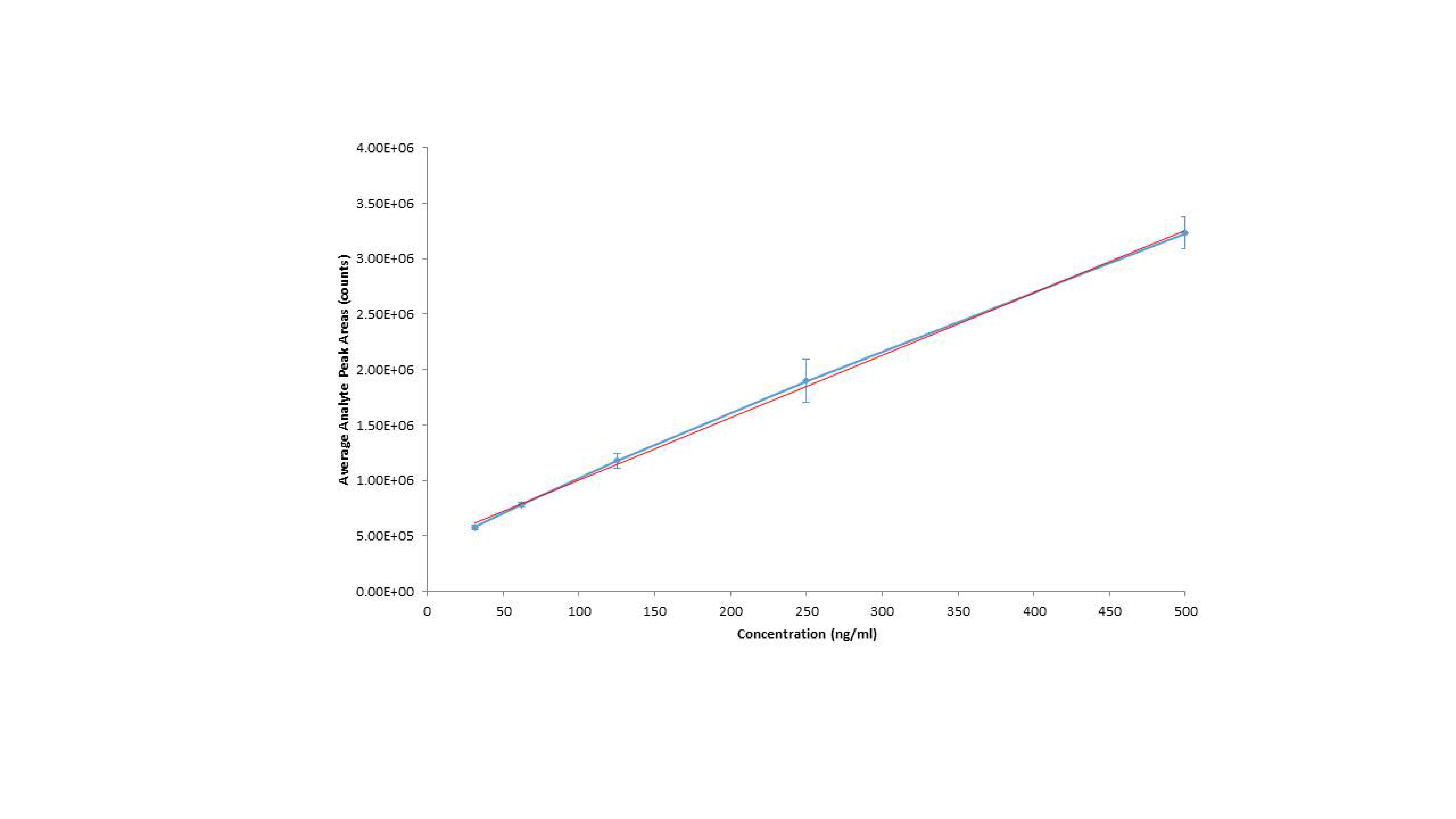

Supplement: S2 Fig — (TIF) [file pone.0141419.s002.tif]

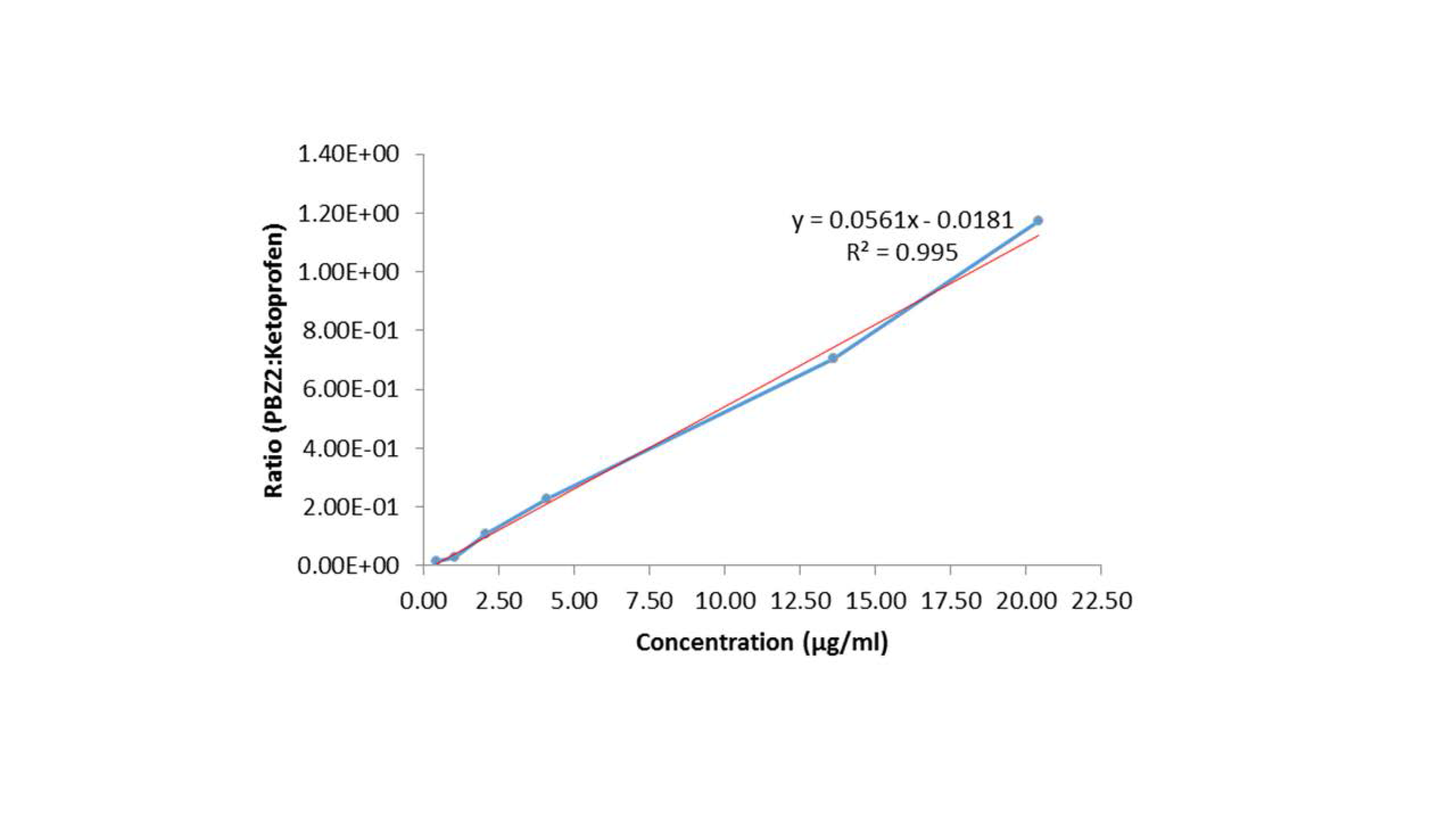

Supplement: S3 Fig — (TIF) [file pone.0141419.s003.tif]
