# Supplementary material for: The Safety and Pharmacokinetics of Carprofen, Flunixin and Phenylbutazone in the Cape Vulture (Gyps coprotheres) following Oral Exposure
Source: PLoS One. 2015 Oct 29;10(10):e0141419. doi: 10.1371/journal.pone.0141419 (PMC4626400; doi:10.1371/journal.pone.0141419)
Supplement: S1 Method — (DOCX) [file pone.0141419.s004.docx]

## S1 Method: Quantification of drug in the plasma

The quantitative data for the different analytes for pharmacokinetic analysis was determined using a liquid chromatography mass spectrometry mass spectrometry (LC/MS-MS) system at the Department of Pharmacology, Faculty of Health Sciences, University of Pretoria utilising an Applied Biosystems/MDS Sciex 4000 Q Trap mass spectrometer with a “Turbo V” ion spray source (electrospray ionisation source ESI); Agilent 1100 series High Pressure Liquid Chromatograph system with a temperature controlled autosampler and six port switching valve; Shimadzu Prominence liquid chromatography LC-20AT and the Analyst 1.5.2 Software package.
