# Supplementary material for: The Safety and Pharmacokinetics of Carprofen, Flunixin and Phenylbutazone in the Cape Vulture (Gyps coprotheres) following Oral Exposure
Source: PLoS One. 2015 Oct 29;10(10):e0141419. doi: 10.1371/journal.pone.0141419 (PMC4626400; doi:10.1371/journal.pone.0141419)
Supplement: S2 Method — (DOCX) [file pone.0141419.s005.docx]

## S2 Method: Carprofen & Flunixin

LC was carried out using the Agilent 1100 series High Pressure Liquid Chromatograph system with a temperature controlled autosampler . Plasma samples were thawed and 200 ul aliquots were diluted 1:1 with water and 25 ul meloxicam (2 ug / ml) was added as an internal, centrifuged at 850G’s for 5 minutes and placed into the autosampler vials. From the temperature controlled (set point of 12°C) auto-sampler a volume of 10 ul, was injected and transferred to the capture column [BDS 10 x 4.6 mm C18], washed with 0.01% formic acid in water at a reduced flow rate for 3.2 minutes and eluted onto a Hypersil C18 DB analytical column [50x4.6 mm]. Elution was achieved with 0.1% formic acid in water at pH 3.1 and resolved with an increasing gradient of 0.1% Formic acid in MeCN before entering the mass spectrometer 4000QTrapmass spectrometer (Applied Biosystems/MDS Sciex) with “Turbo V” ion spray source. The mass spectrometer was set on a negative polarity mode, MRM scan type and a unit resolution for both Q1 and Q3.The ionisation source voltage was -4000.00 V, extraction potential -10.00 V and collision cell extraction potential -10.00 V. Tuning parameters for carprofen 1 were Q1 mass of 272.3 Da; Q3 mass of 228.0 Da; 80 msec Dwell time; declustering potential (DP) -40.00 and collision energy (CE) of -32.00. Carprofen 2 parameters differed with a Q3 mass of 190.1 Da and CE -55.00. Tuning parameters for flunixin 1 were Q1 mass of 295.4 Da; Q3 mass of 251.2.0 Da; 80 msec Dwell time; DP -35.00 and CE of -28.00. Flunixin 2 parameters differed with a Q3 mass of 209.3 Da and CE -35.00. Tuning parameters for Meloxicam 1 were Q1 mass of 350.4 Da; Q3 mass of 146.2 Da; 80 msec Dwell time; DP -35.00 and CE of -30.00. Meloxicam 2 parameters differed by Q3 mass of 286.4 and CE of -20.00.

**Calibration Curves:** Freshly drawn chicken plasma was spiked with the different analytes at seven different concentrations covering the expected concentrations to be found in the vulture plasma samples to create calibration curves. Three concentration series of 31.3, 62.5, 125, 250, 500, 1000 and 2000 ng/ml of each standard were made and analysed separately (run 1 – 3). A linear calibration curve (R^2^ = 0.9979) for carprofen was evident over the concentration range of 31.3 – 2000 ng/ml (Figure S-1). Flunixin calibration curve was linear (R^2^ = 0.9987) over the concentration range of 31.3 – 500 ng/ml (Figure S-2). The signal to noise ratio at the lowest concentration of the curve was 28.9 and 25.8 (Analyst software v.1.5.2) for carprofen and flunixin respectively. The regression line slope was utilised as the response factor. The value of the zero hour sample for each bird was subtracted from each subsequent time point’s concentration value to account for background noise.
