# Supplementary material for: The Safety and Pharmacokinetics of Carprofen, Flunixin and Phenylbutazone in the Cape Vulture (Gyps coprotheres) following Oral Exposure
Source: PLoS One. 2015 Oct 29;10(10):e0141419. doi: 10.1371/journal.pone.0141419 (PMC4626400; doi:10.1371/journal.pone.0141419)
Supplement: S3 Method — (DOCX) [file pone.0141419.s006.docx]

## S3 Method: Phenylbutazone (PBZ) analysis

Manual extraction was performed due to low recovery when using the capture column technique. Methanol/Ketoprofen, 100 ul (54 ug/ml ketoprofen in methanol), was added to 100 ul aliquots of thawed plasma and sonicated for 10 minutes in the ultrasonic bath. A further 100 ul methanol was added followed by 10 minutes sonication. The sample was centrifuged for 10 minutes at 14800 rpm (Beckman Coulter Microfuge 16 centrifuge) and 100 ul of the supernatant was transferred into autosampler vials. From the temperature controlled (set point of 12°C) auto-sampler a volume of 10 ul, was injected onto a Hypersil C18 DB analytical column [50 x 4.6 mm]. The mobile phase consisted of A: 0.1% Formic acid in water at a pH of 6.1; B: 5mM Ammonium formate in 27% water: acetonitrile at a pH of 6.1. Gradient elution was performed using the following gradient (total time indicated): 0 – 0 min, 75% A; 1 – 0.75 min, 75%A; 2 – 4 min, 5% A; 3 – 5 min, 5% A; 4 – 5.5.min, 75% A; 5 – 7 min, 75% A. The sample was passed through a diode array detector and into the mass spectrometer. Mass spectrometer was set on a negative polarity mode, MRM scan type and a unit resolution for both Q1 and Q3.The ionisation source voltage was -4000.00 V, extraction potential -10.00 V and collision cell extraction potential -10.00 V. Tuning parameters for PBZ 1 were as follows: Q1 mass 307.4 Da; Q3 mass 279.5 Da; 80 ms Dwell time; declustering potential (DP) -35.00 and collision energy (CE) of -28.00. PBZ 2 parameters differed with a Q3 mass of 131 Da and CE -40.00. The diode array with UV lamp on and visible lamp off was set in spectral operating mode, starting at 210 and stopping at 400 with a 2 step width and 100 margin for negative absorbance.

**Calibration curve:** Chicken plasma or solvent was spiked with the phenylbutazone at seven different concentrations (0.41, 1.02, 2.04, 4.08, 10.2, 13.6 & 20.4 ug/ml) covering the expected concentrations to be found in the vulture plasma samples. Ketoprofen was used as an internal standard (IS). The calibration was run twice for the solvent and plasma and plotted in Analyst software. The conversion of peak areas to concentrations was performed using the response factor determined from the calibration curve. A linear relationship for phenylbutazone between concentration and peak area (R^2^ = 0.9948) over the concentration range of 0.41 – 20.4 ug/ml was demonstrated (Figure S-3). The signal to noise ratio at the lowest concentration of the curve was 144.9 (Analyst software).
