# Supplementary material for: The Safety and Pharmacokinetics of Carprofen, Flunixin and Phenylbutazone in the Cape Vulture (Gyps coprotheres) following Oral Exposure
Source: PLoS One. 2015 Oct 29;10(10):e0141419. doi: 10.1371/journal.pone.0141419 (PMC4626400; doi:10.1371/journal.pone.0141419)
Supplement: S1 Table — (DOCX) [file pone.0141419.s007.docx]

| **Table S-1: Mean and standard deviation (SD) of the serum ALB concentrations (g/L) per treatment group per time of sampling.** | | | | | | | | | | | | | | | | | | | |
| --- | --- | --- | --- | --- | --- | --- | --- | --- | --- | --- | --- | --- | --- | --- | --- | --- | --- | --- | --- |
| **Time Point** | **Carprofen** | | | |  | **Flunixin** | | | |  | **Phenylbutazone** | | | |  | **Control** | | | |
|  | **Bird 1** | **Bird 2** | **Mean** | **SD** |  | **Bird 3** | **Bird 4** | **Mean** | **SD** |  | **Bird 5** | **Bird 6** | **Mean** | **SD** |  | **Bird 7** | **Bird 8** | **Mean** | **SD** |
| **0 h** | 15.20 | 11.40 | 13.30 | 2.69 |  | 15.00 | 13.50 | 14.25 | 1.06 |  | 11.90 | 14.50 | 13.20 | 1.84 |  | 10.00 | 14.10 | 12.05 | 2.90 |
| **0.5 h** | 15.10 | 11.60 | 13.35 | 2.47 |  | 15.00 | 13.30 | 14.15 | 1.20 |  | 11.60 | 13.80 | 12.70 | 1.56 |  | 9.60 | 13.30 | 11.45 | 2.62 |
| **1 h** | 15.00 | 11.50 | 13.25 | 2.47 |  | 14.60 | 13.10 | 13.85 | 1.06 |  | 11.30 | 13.00 | 12.15 | 1.20 |  | 9.80 | 14.10 | 11.95 | 3.04 |
| **1.5 h** | 14.40 | 11.10 | 12.75 | 2.33 |  | 14.60 | 13.00 | 13.80 | 1.13 |  | 11.10 | 13.40 | 12.25 | 1.63 |  | 9.70 | NS | 9.70 |  |
| **2 h** | 13.90 | NS | 13.90 |  |  | 14.30 | 12.60 | 13.45 | 1.20 |  | 11.20 | 13.10 | 12.15 | 1.34 |  | 9.10 | 13.10 | 11.10 | 2.83 |
| **3 h** | NS | 11.70 | 11.70 |  |  | 14.40 | 12.90 | 13.65 | 1.06 |  | 10.60 | 11.40 | 11.00 | 0.57 |  | 9.30 | 12.80 | 11.05 | 2.47 |
| **5 h** | 12.90 | 10.10 | 11.50 | 1.98 |  | 13.90 | 12.80 | 13.35 | 0.78 |  | 11.50 | 11.80 | 11.65 | 0.21 |  | 9.30 | NS | 9.30 |  |
| **7 h** | 12.30 | 10.80 | 11.55 | 1.06 |  | 13.70 | 13.00 | 13.35 | 0.49 |  | 11.50 | NS | 11.50 |  |  | 8.80 | 11.50 | 10.15 | 1.91 |
| **9 h** | 9.60 | 10.30 | 9.95 | 0.49 |  | 13.40 | 13.00 | 13.20 | 0.28 |  | 12.90 | NS | 12.90 |  |  | 9.00 | 5.50 | 7.25 | 2.47 |
| **12 h** | 12.90 | NS | 12.90 |  |  | 13.80 | 12.20 | 13.00 | 1.13 |  | 10.40 | NS | 10.40 |  |  | 9.40 | 16.70 | 13.05 | 5.16 |
| **24 h** | 13.40 | 10.70 | 12.05 | 1.91 |  | 12.60 | 12.70 | 12.65 | 0.07 |  | 13.00 | NS | 13.00 |  |  | 9.80 | 10.90 | 10.35 | 0.78 |
| **32 h** | NS | 11.30 | 11.30 |  |  | 13.00 | 13.30 | 13.15 | 0.21 |  | 11.80 | 11.50 | 11.65 | 0.21 |  | 10.00 | 10.30 | 10.15 | 0.21 |
| **48 h** | NS | 13.60 | 13.60 |  |  | 13.70 | 14.60 | 14.15 | 0.64 |  | 13.50 | 13.40 | 13.45 | 0.07 |  | 11.60 | 12.20 | 11.90 | 0.42 |
| NS – No sample. Reference values: ALB 9.46 – 17.31 g/l | | | | | | | | | | | | | | | |  |  |  |  |
