# Supplementary material for: The Safety and Pharmacokinetics of Carprofen, Flunixin and Phenylbutazone in the Cape Vulture (Gyps coprotheres) following Oral Exposure
Source: PLoS One. 2015 Oct 29;10(10):e0141419. doi: 10.1371/journal.pone.0141419 (PMC4626400; doi:10.1371/journal.pone.0141419)
Supplement: S2 Table — (DOCX) [file pone.0141419.s008.docx]

| **Table S-2: Mean and standard deviation (SD) of the serum activities of ALT (U/L) per treatment group per time of sampling.** | | | | | | | | | | | | | | | | | | | |
| --- | --- | --- | --- | --- | --- | --- | --- | --- | --- | --- | --- | --- | --- | --- | --- | --- | --- | --- | --- |
| **Time Point** | **Carprofen** | | | |  | **Flunixin** | | | |  | **Phenylbutazone** | | | |  | **Control** | | | |
|  | **Bird 1** | **Bird 2** | **Mean** | **SD** |  | **Bird 3** | **Bird 4** | **Mean** | **SD** |  | **Bird 5** | **Bird 6** | **Mean** | **SD** |  | **Bird 7** | **Bird 8** | **Mean** | **SD** |
| **0 h** | 36.00 | 18.00 | 27.00 | 12.73 |  | 27.00 | 25.00 | 26.00 | 1.41 |  | 13.00 | 13.00 | 13.00 | 0.00 |  | 9.00 | 21.00 | 15.00 | 8.49 |
| **0.5 h** | 34.00 | 15.00 | 24.50 | 13.44 |  | 30.00 | 30.00 | 30.00 | 0.00 |  | 24.00 | 26.00 | 25.00 | 1.41 |  | 24.00 | 20.00 | 22.00 | 2.83 |
| **1 h** | 29.00 | 17.00 | 23.00 | 8.49 |  | 20.00 | 40.00 | 30.00 | 14.14 |  | 14.00 | 38.00 | 26.00 | 16.97 |  | 8.00 | 19.00 | 13.50 | 7.78 |
| **1.5 h** | 32.00 | 13.00 | 22.50 | 13.44 |  | 24.00 | 29.00 | 26.50 | 3.54 |  | 22.00 | 32.00 | 27.00 | 7.07 |  | 8.00 | NS | 8.00 |  |
| **2 h** | 33.00 | NS | 33.00 |  |  | 30.00 | 34.00 | 32.00 | 2.83 |  | 16.00 | 31.00 | 23.50 | 10.61 |  | 6.00 | 16.00 | 11.00 | 7.07 |
| **3 h** | NS | 14.00 | 14.00 |  |  | 27.00 | 36.00 | 31.50 | 6.36 |  | 38.00 | 29.00 | 33.50 | 6.36 |  | 31.00 | 36.00 | 33.50 | 3.54 |
| **5 h** | 45.00 | 25.00 | 35.00 | 14.14 |  | 30.00 | 59.00 | 44.50 | 20.51 |  | 29.00 | 55.00 | 42.00 | 18.38 |  | 16.00 | NS | 16.00 |  |
| **7 h** | 61.00 | 48.00 | 54.50 | 9.19 |  | 41.00 | 70.00 | 55.50 | 20.51 |  | 39.00 | NS | 39.00 |  |  | 7.00 | 28.00 | 17.50 | 14.85 |
| **9 h** | 111.00 | 65.00 | 88.00 | 32.53 |  | 36.00 | 128.00 | 82.00 | 65.05 |  | 47.00 | NS | 47.00 |  |  | 46.00 | 117.00 | 81.50 | 50.20 |
| **12 h** | 124.00 | NS | 124.00 |  |  | 52.00 | 156.00 | 104.00 | 73.54 |  | 54.00 | NS | 54.00 |  |  | 36.00 | 61.00 | 48.50 | 17.68 |
| **24 h** | 103.00 | 77.00 | 90.00 | 18.38 |  | 53.00 | 157.00 | 105.00 | 73.54 |  | 54.00 | NS | 54.00 |  |  | 33.00 | 73.00 | 53.00 | 28.28 |
| **32 h** | NS | 58.00 | 58.00 |  |  | 58.00 | 264.00 | 161.00 | 145.66 |  | 59.00 | 78.00 | 68.50 | 13.44 |  | 36.00 | 33.00 | 34.50 | 2.12 |
| **48 h** | NS | 44.00 | 44.00 |  |  | 34.00 | 246.00 | 140.00 | 149.91 |  | 62.00 | 83.00 | 72.50 | 14.85 |  | 21.00 | 43.00 | 32.00 | 15.56 |
| NS – No sample. Reference values: ALT 31.2 – 60.1 u/l | | | | | | | | | | | | | | | |  |  |  |  |
