# Supplementary material for: The Safety and Pharmacokinetics of Carprofen, Flunixin and Phenylbutazone in the Cape Vulture (Gyps coprotheres) following Oral Exposure
Source: PLoS One. 2015 Oct 29;10(10):e0141419. doi: 10.1371/journal.pone.0141419 (PMC4626400; doi:10.1371/journal.pone.0141419)
Supplement: S3 Table — (DOCX) [file pone.0141419.s009.docx]

| **Table S-3: Mean and standard deviation (SD) of the serum Ca2+ concentrations (mmol/l) per treatment group per time of sampling.** | | | | | | | | | | | | | | | | | | | |
| --- | --- | --- | --- | --- | --- | --- | --- | --- | --- | --- | --- | --- | --- | --- | --- | --- | --- | --- | --- |
| **Time Point** | **Carprofen** | | | |  | **Flunixin** | | | |  | **Phenylbutazone** | | | |  | **Control** | | | |
|  | **Bird 1** | **Bird 2** | **Mean** | **SD** |  | **Bird 3** | **Bird 4** | **Mean** | **SD** |  | **Bird 5** | **Bird 6** | **Mean** | **SD** |  | **Bird 7** | **Bird 8** | **Mean** | **SD** |
| **0 h** | 2.53 | 2.00 | 2.27 | 0.37 |  | 2.21 | 1.94 | 2.08 | 0.19 |  | 2.10 | 2.78 | 2.44 | 0.48 |  | 2.02 | 2.14 | 2.08 | 0.08 |
| **0.5 h** | 2.52 | 1.95 | 2.24 | 0.40 |  | 2.43 | 1.96 | 2.20 | 0.33 |  | 1.84 | 2.50 | 2.17 | 0.47 |  | 1.39 | 2.20 | 1.80 | 0.57 |
| **1 h** | 2.09 | 1.97 | 2.03 | 0.08 |  | 2.25 | 1.71 | 1.98 | 0.38 |  | 1.93 | 2.06 | 2.00 | 0.09 |  | 1.99 | 2.29 | 2.14 | 0.21 |
| **1.5 h** | 2.40 | 2.00 | 2.20 | 0.28 |  | 2.14 | 1.86 | 2.00 | 0.20 |  | 1.80 | 2.50 | 2.15 | 0.49 |  | 1.76 | NS | 1.76 |  |
| **2 h** | 2.32 | NS | 2.32 |  |  | 2.29 | 2.02 | 2.16 | 0.19 |  | 1.92 | 2.49 | 2.21 | 0.40 |  | 1.67 | 2.02 | 1.85 | 0.25 |
| **3 h** | NS | 1.97 | 1.97 |  |  | 2.23 | 1.85 | 2.04 | 0.27 |  | 1.58 | 2.03 | 1.81 | 0.32 |  | 1.52 | 1.61 | 1.57 | 0.06 |
| **5 h** | 2.31 | 1.79 | 2.05 | 0.37 |  | 2.09 | 1.99 | 2.04 | 0.07 |  | 1.77 | 1.93 | 1.85 | 0.11 |  | 1.66 | NS | 1.66 |  |
| **7 h** | 1.88 | 1.53 | 1.71 | 0.25 |  | 2.18 | 1.90 | 2.04 | 0.20 |  | 1.55 | NS | 1.55 |  |  | 0.84 | 1.14 | 0.99 | 0.21 |
| **9 h** | 1.46 | 1.27 | 1.37 | 0.13 |  | 2.04 | 1.66 | 1.85 | 0.27 |  | 1.86 | NS | 1.86 |  |  | 1.51 | 1.19 | 1.35 | 0.23 |
| **12 h** | 1.67 | NS | 1.67 |  |  | 1.48 | 1.18 | 1.33 | 0.21 |  | 1.24 | NS | 1.24 |  |  | 1.36 | 1.65 | 1.51 | 0.21 |
| **24 h** | 2.07 | 1.26 | 1.67 | 0.57 |  | 2.07 | 2.02 | 2.05 | 0.04 |  | 1.80 | NS | 1.80 |  |  | 1.47 | 1.47 | 1.47 | 0.00 |
| **32 h** | NS | 1.47 | 1.47 |  |  | 2.05 | 1.90 | 1.98 | 0.11 |  | 1.43 | 2.23 | 1.83 | 0.57 |  | 1.78 | 1.49 | 1.64 | 0.21 |
| **48 h** | NS | 2.10 | 2.10 |  |  | 2.03 | 2.02 | 2.03 | 0.01 |  | 1.92 | 2.19 | 2.06 | 0.19 |  | 2.09 | 1.40 | 1.75 | 0.49 |
| NS – No sample. Reference values: Ca2+ 0.44 – 1.35 mmol/l | | | | | | | | | | | | | | | |  |  |  |  |
