# Supplementary material for: The Safety and Pharmacokinetics of Carprofen, Flunixin and Phenylbutazone in the Cape Vulture (Gyps coprotheres) following Oral Exposure
Source: PLoS One. 2015 Oct 29;10(10):e0141419. doi: 10.1371/journal.pone.0141419 (PMC4626400; doi:10.1371/journal.pone.0141419)
Supplement: S4 Table — (DOCX) [file pone.0141419.s010.docx]

**Table S-4: Mean and standard deviation (SD) of the serum Na concentrations (mmol/l) per treatment group per time of sampling.**

| **Time Point** | **Carprofen** | | | |  | **Flunixin** | | | | |  | | **Phenylbutazone** | | | | |  | | **Control** | | | | | | | |
| --- | --- | --- | --- | --- | --- | --- | --- | --- | --- | --- | --- | --- | --- | --- | --- | --- | --- | --- | --- | --- | --- | --- | --- | --- | --- | --- | --- |
|  | **Bird 1** | **Bird 2** | **Mean** | **SD** |  | **Bird 3** | **Bird 4** | **Mean** | **SD** |  | | **Bird 5** | | **Bird 6** | **Mean** | **SD** |  | | **Bird 7** | | **Bird 8** | | **Mean** | | **SD** | |  |
| **0 h** | 151.00 | 149.70 | 150.35 | 0.92 |  | 149.60 | 151.70 | 150.65 | 1.48 |  | | 146.80 | | 155.90 | 151.35 | 6.43 |  | | 150.20 | | 146.20 | | 148.20 | | 2.83 | |  |
| **0.5 h** | 149.70 | 151.40 | 150.55 | 1.20 |  | 146.80 | 152.30 | 149.55 | 3.89 |  | | 145.30 | | 148.20 | 146.75 | 2.05 |  | | 149.10 | | 147.00 | | 148.05 | | 1.48 | |  |
| **1 h** | 151.20 | 150.00 | 150.60 | 0.85 |  | 144.40 | 148.80 | 146.60 | 3.11 |  | | 146.30 | | 135.20 | 140.75 | 7.85 |  | | 151.00 | | 150.20 | | 150.60 | | 0.57 | |  |
| **1.5 h** | 149.00 | 149.00 | 149.00 | 0.00 |  | 145.40 | 150.20 | 147.80 | 3.39 |  | | 143.50 | | 150.60 | 147.05 | 5.02 |  | | 151.60 | | NS | | 151.60 | |  | |  |
| **2 h** | 150.40 | NS | 150.40 |  |  | 144.70 | 150.50 | 147.60 | 4.10 |  | | 146.10 | | 150.60 | 148.35 | 3.18 |  | | 150.10 | | 144.60 | | 147.35 | | 3.89 | |  |
| **3 h** | NS | 148.60 | 148.60 |  |  | 147.00 | 151.70 | 149.35 | 3.32 |  | | 144.30 | | 145.80 | 145.05 | 1.06 |  | | 149.70 | | 140.60 | | 145.15 | | 6.43 | |  |
| **5 h** | 148.70 | 143.90 | 146.30 | 3.39 |  | 144.20 | 152.10 | 148.15 | 5.59 |  | | 145.50 | | 146.20 | 145.85 | 0.49 |  | | 149.20 | | NS | | 149.20 | |  | |  |
| **7 h** | 147.00 | 146.30 | 146.65 | 0.49 |  | 146.10 | 159.90 | 153.00 | 9.76 |  | | 145.20 | | NS | 145.20 |  |  | | 148.40 | | 123.60 | | 136.00 | | 17.54 | |  |
| **9 h** | 125.50 | 137.80 | 131.65 | 8.70 |  | 140.00 | 143.20 | 141.60 | 2.26 |  | | 156.10 | | NS | 156.10 |  |  | | 146.80 | | 104.80 | | 125.80 | | 29.70 | |  |
| **12 h** | 145.20 | NS | 145.20 |  |  | 142.90 | 144.60 | 143.75 | 1.20 |  | | 125.60 | | NS | 125.60 |  |  | | 142.10 | | 143.60 | | 142.85 | | 1.06 | |  |
| **24 h** | 144.30 | 139.70 | 142.00 | 3.25 |  | 141.30 | 146.50 | 143.90 | 3.68 |  | | 144.60 | | NS | 144.60 |  |  | | 142.30 | | 123.60 | | 132.95 | | 13.22 | |  |
| **32 h** | NS | 141.10 | 141.10 |  |  | 140.40 | 143.80 | 142.10 | 2.40 |  | | 141.40 | | 143.50 | 142.45 | 1.48 |  | | 143.20 | | 109.70 | | 126.45 | | 23.69 | |  |
| **48 h** | NS | 151.80 | 151.80 |  |  | 144.30 | 146.70 | 145.50 | 1.70 |  | | 150.00 | | 147.70 | 148.85 | 1.63 |  | | 147.50 | | 123.20 | | 135.35 | | 17.18 | |  |
| NS – No sample. Reference values: Na 136.36 – 149.45 mmol/l | | | | | | | | | | | | | | | | | | | |  | |  | |  | |  | |
