# Supplementary material for: The Safety and Pharmacokinetics of Carprofen, Flunixin and Phenylbutazone in the Cape Vulture (Gyps coprotheres) following Oral Exposure
Source: PLoS One. 2015 Oct 29;10(10):e0141419. doi: 10.1371/journal.pone.0141419 (PMC4626400; doi:10.1371/journal.pone.0141419)
Supplement: S5 Table — (DOCX) [file pone.0141419.s011.docx]

**Table S-5: Mean and standard deviation (SD) of the serum K^=^ concentrations (mmol/l) per treatment group per time of sampling.**

| **Time Point** | **Carprofen** | | | |  | **Flunixin** | | | |  | **Phenylbutazone** | | | |  | **Control** | | | |
| --- | --- | --- | --- | --- | --- | --- | --- | --- | --- | --- | --- | --- | --- | --- | --- | --- | --- | --- | --- |
|  | **Bird 1** | **Bird 2** | **Mean** | **SD** |  | **Bird 3** | **Bird 4** | **Mean** | **SD** |  | **Bird 5** | **Bird 6** | **Mean** | **SD** |  | **Bird 7** | **Bird 8** | **Mean** | **SD** |
| **0 h** | 3.47 | 3.80 | 3.64 | 0.23 |  | 3.06 | 3.38 | 3.22 | 0.23 |  | 3.14 | 2.72 | 2.93 | 0.30 |  | 3.03 | 2.84 | 2.94 | 0.13 |
| **0.5 h** | 2.96 | 3.42 | 3.19 | 0.33 |  | 2.36 | 2.91 | 2.64 | 0.39 |  | 3.32 | 3.20 | 3.26 | 0.08 |  | 4.27 | 2.45 | 3.36 | 1.29 |
| **1 h** | 2.72 | 4.48 | 3.60 | 1.24 |  | 3.06 | 6.83 | 4.95 | 2.67 |  | 2.79 | 4.28 | 3.54 | 1.05 |  | 2.97 | 2.83 | 2.90 | 0.10 |
| **1.5 h** | 2.78 | 3.29 | 3.04 | 0.36 |  | 4.16 | 4.25 | 4.21 | 0.06 |  | 3.91 | 2.90 | 3.41 | 0.71 |  | 3.75 | NS | 3.75 |  |
| **2 h** | 2.47 | NS | 2.47 |  |  | 3.38 | 3.97 | 3.68 | 0.42 |  | 2.60 | 3.24 | 2.92 | 0.45 |  | 3.09 | 3.50 | 3.30 | 0.29 |
| **3 h** | NS | 1.68 | 1.68 |  |  | 1.50 | 2.38 | 1.94 | 0.62 |  | 5.39 | 5.91 | 5.65 | 0.37 |  | 4.50 | 5.91 | 5.21 | 1.00 |
| **5 h** | 2.67 | 4.00 | 3.34 | 0.94 |  | 3.66 | 4.42 | 4.04 | 0.54 |  | 3.07 | 8.57 | 5.82 | 3.89 |  | 5.34 | NS | 5.34 |  |
| **7 h** | 3.43 | 6.46 | 4.95 | 2.14 |  | 3.07 | 3.29 | 3.18 | 0.16 |  | 3.12 | NS | 3.12 |  |  | 3.34 | 3.22 | 3.28 | 0.08 |
| **9 h** | 13.44 | 10.86 | 12.15 | 1.82 |  | 3.94 | 5.70 | 4.82 | 1.24 |  | 3.77 | NS | 3.77 |  |  | 6.04 | 20.97 | 13.51 | 10.56 |
| **12 h** | 6.49 | NS | 6.49 |  |  | 4.13 | 5.58 | 4.86 | 1.03 |  | 4.68 | NS | 4.68 |  |  | 5.63 | 5.16 | 5.40 | 0.33 |
| **24 h** | 2.74 | 7.61 | 5.18 | 3.44 |  | 3.46 | 3.78 | 3.62 | 0.23 |  | 3.72 | NS | 3.72 |  |  | 4.52 | 7.23 | 5.88 | 1.92 |
| **32 h** | NS | 3.27 | 3.27 |  |  | 3.09 | 3.14 | 3.12 | 0.04 |  | 3.53 | 3.83 | 3.68 | 0.21 |  | 3.98 | 2.80 | 3.39 | 0.83 |
| **48 h** | NS | 3.30 | 3.30 |  |  | 1.80 | 2.14 | 1.97 | 0.24 |  | 2.38 | 4.85 | 3.62 | 1.75 |  | 3.15 | 2.31 | 2.73 | 0.59 |
| NS – No sample. Reference values: K 1.49 – 7.15 mmol/l | | | | | | | | | | | | | | | |  |  |  |  |
