# Supplementary material for: The Safety and Pharmacokinetics of Carprofen, Flunixin and Phenylbutazone in the Cape Vulture (Gyps coprotheres) following Oral Exposure
Source: PLoS One. 2015 Oct 29;10(10):e0141419. doi: 10.1371/journal.pone.0141419 (PMC4626400; doi:10.1371/journal.pone.0141419)
Supplement: S6 Table — (DOCX) [file pone.0141419.s012.docx]

**Table S-6: Mean and standard deviation (SD) of the serum UA concentrations (mmol/l) per treatment group per time of sampling.**

|  | | | | | | | | | | | | | | | | | | | |
| --- | --- | --- | --- | --- | --- | --- | --- | --- | --- | --- | --- | --- | --- | --- | --- | --- | --- | --- | --- |
| **Time Point** | **Carprofen** | | | |  | **Flunixin** | | | |  | **Phenylbutazone** | | | |  | **Control** | | | |
|  | **Bird 1** | **Bird 2** | **Mean** | **SD** |  | **Bird 3** | **Bird 4** | **Mean** | **SD** |  | **Bird 5** | **Bird 6** | **Mean** | **SD** |  | **Bird 7** | **Bird 8** | **Mean** | **SD** |
| **0 h** | 0.47 | 0.46 | 0.47 | 0.01 |  | 0.29 | 0.50 | 0.40 | 0.15 |  | 0.28 | 0.26 | 0.27 | 0.01 |  | 0.32 | 0.24 | 0.28 | 0.06 |
| **0.5 h** | 0.72 | 0.58 | 0.65 | 0.10 |  | 0.45 | 0.62 | 0.54 | 0.12 |  | 0.34 | 0.34 | 0.34 | 0.00 |  | 0.22 | 0.25 | 0.24 | 0.02 |
| **1 h** | 0.71 | 0.60 | 0.66 | 0.08 |  | 0.35 | 0.52 | 0.44 | 0.12 |  | 0.37 | 0.30 | 0.34 | 0.05 |  | 0.30 | 0.27 | 0.29 | 0.02 |
| **1.5 h** | 0.60 | 0.54 | 0.57 | 0.04 |  | 0.42 | 0.83 | 0.63 | 0.29 |  | 0.45 | 0.36 | 0.41 | 0.06 |  | 0.34 | NS | 0.34 |  |
| **2 h** | 0.65 | NS | 0.65 |  |  | 0.39 | 0.70 | 0.55 | 0.22 |  | 0.47 | 0.33 | 0.40 | 0.10 |  | 0.33 | 0.20 | 0.27 | 0.09 |
| **3 h** | NS | 0.60 | 0.60 |  |  | 0.36 | 0.84 | 0.60 | 0.34 |  | 0.49 | 0.49 | 0.49 | 0.00 |  | 0.35 | 0.17 | 0.26 | 0.13 |
| **5 h** | 0.57 | 0.54 | 0.56 | 0.02 |  | 0.37 | 1.11 | 0.74 | 0.52 |  | 0.53 | 0.37 | 0.45 | 0.11 |  | 0.35 | NS | 0.35 |  |
| **7 h** | 0.40 | 0.64 | 0.52 | 0.17 |  | 0.33 | 0.80 | 0.57 | 0.33 |  | 0.49 | NS | 0.49 |  |  | 0.37 | 0.18 | 0.28 | 0.13 |
| **9 h** | 0.36 | 0.59 | 0.48 | 0.16 |  | 0.34 | 0.52 | 0.43 | 0.13 |  | 0.57 | NS | 0.57 |  |  | 0.34 | 0.08 | 0.21 | 0.18 |
| **12 h** | 0.53 | NS | 0.53 |  |  | 0.34 | 0.40 | 0.37 | 0.04 |  | 0.50 | NS | 0.50 |  |  | 0.24 | 0.13 | 0.19 | 0.08 |
| **24 h** | 0.37 | 0.41 | 0.39 | 0.03 |  | 0.28 | 0.39 | 0.34 | 0.08 |  | 0.36 | NS | 0.36 |  |  | 0.21 | 0.14 | 0.18 | 0.05 |
| **32 h** | NS | 0.24 | 0.24 |  |  | 0.25 | 0.42 | 0.34 | 0.12 |  | 0.33 | 0.43 | 0.38 | 0.07 |  | 0.22 | 0.10 | 0.16 | 0.08 |
| **48 h** | NS | 0.18 | 0.18 |  |  | 0.22 | 0.35 | 0.29 | 0.09 |  | 0.35 | 0.30 | 0.33 | 0.04 |  | 0.18 | 0.10 | 0.14 | 0.06 |
| NS – No sample. Reference values: UA 0.15 – 0.65 mmol/l | | | | | | | | | | | | | | | |  |  |  |  |
